# “Capacity Building in Community-Engaged Research: The Flagstaff Dementia-Friendly City Initiative”

**DOI:** 10.1093/geroni/igaf122.714

**Published:** 2025-12-31

**Authors:** Eric Cerino, Michael McCarthy, Charlton Wilson, Terry Smith, Karin Von Kay, Cindy Martin, Mary Gemma O’Donnell

**Affiliations:** Northern Arizona University, Flagstaff, Arizona, United States; Northern Arizona University, Flagstaff, Arizona, United States; Northern Arizona Regional Behavioral Health Authority Institute, Flagstaff, Arizona, United States; Northern Arizona University, Flagstaff, Arizona, United States; Northern Arizona Alzheimer’s and Dementia Alliance, Flagstaff, Arizona, United States; Northern Arizona Regional Behavioral Health Authority Institute, Flagstaff, Arizona, United States; Northern Arizona University, Flagstaff, Arizona, United States

## Abstract

**Research Aims & Significance:**

The Flagstaff Dementia-Friendly City Initiative demonstrates how capacity building in community-engaged research can drive sustainable change. While research was not the initial goal, the initiative naturally integrated Community-Based Participatory Research (CBPR) principles, strengthening local research capacity while addressing dementia as a growing public health challenge in northern Arizona.

**Methods & Approach:**

Collaborative partnerships among Northern Arizona University (NAU), the Northern Arizona Regional Behavioral Health Authority Institute (NARBHA), the Northern Arizona Alzheimer’s and Dementia Alliance (NAZADA), caregivers, people with memory challenges, healthcare providers, and local organizations formed the foundation for an inclusive framework. Using the Dementia Friendly America Community Toolkit, the initiative followed a four-phase process: Convene, Engage, Analyze, and Act, leading to the formation of a 30+ member Dementia-Friendly Community Council representing diverse sectors.

**Key Findings:**

Capacity-building strategies included establishing collaborative governance structures, conducting a community needs assessment, and expanding dementia-friendly education programs. Within a year, Flagstaff achieved Dementia-Friendly City designation, integrating research-driven frameworks into community programs, public service improvements, and caregiver respite initiatives.

**Implications for Research, Policy & Practice:**

Challenges such as sustaining engagement and aligning research priorities with community needs highlighted the necessity of distributed leadership and ongoing relationship-building. Future directions focus on broadening dementia-friendly policies, increasing community education and respite care resources, and enhancing accessibility across public and healthcare spaces. This initiative illustrates how CBPR enhances local research capacity while informing dementia-friendly policy and services, offering a model for other communities pursuing similar designations.

